# ﻿Description of a new *Osedax* (Annelida, Polychaeta, Siboglinidae) species colonizing cow bones in the South Atlantic Ocean

**DOI:** 10.3897/zookeys.1219.134005

**Published:** 2024-12-03

**Authors:** Thammy Gularte, Paulo Y. G. Sumida, Gilberto Bergamo, Greg W. Rouse

**Affiliations:** 1 Biological Oceanography Department, Oceanographic Institute – University of São Paulo, Praça do Oceanográfico, 191, 05508-120, São Paulo, SP, Brazil University of São Paulo São Paulo Brazil; 2 Scripps Institution of Oceanography, UC San Diego, La Jolla CA, 92093-0202, USA Scripps Institution of Oceanography La Jolla United States of America

**Keywords:** New species, phylogeny, taxonomy, whale falls

## Abstract

A new species of *Osedax* is described here using molecular and morphological data. It was found at the depth of 550 m off the Brazilian coast through experimental deployment of cow bones. *Osedaxnataliae***sp. nov.** is the second *Osedax* species from the Southwest Atlantic Ocean and had been previously reported as *Osedax* ‘BioSuOr-4’. Phylogenetic analysis of five concatenated genetic makers (28S rDNA, Histone H3, 18S rDNA, 16S rDNA, and cytochrome c oxidase I) placed *Osedaxnataliae***sp. nov.** within a well-supported *Osedax* Clade V, nested within a clade of Pacific Ocean *Osedax* though with poor support. The minimum interspecific COI distance between *O.nataliae***sp. nov.** and another known *Osedax* was 13.92% (closest to *O.* ‘sagami-3’). The maximum intraspecific COI diversity (uncorrected) within *O.nataliae***sp. nov.** sampled here was 2.44% and population structure was visualized via haplotype network analysis. Morphologically, *O.nataliae***sp. nov.** is characterized by its reddish orange crown of palps and a ventral yellowish collar on the anterior trunk where it meets the base of the crown. *Osedaxnataliae***sp. nov.** shares features with other Clade V species, notably pinnules inserted on the outer margin of palps. Additionally, the presence of dwarf males within the tube lumen of females was documented. Further sampling and research in the Southern Hemisphere are needed to understand the diversity and biogeography of *Osedax* across the world’s oceans.

## ﻿Introduction

Dead whale carcasses are one of the most remarkable energy sources for a plethora of deep-sea organisms as they sink and reach the deep seafloor, known as whale falls, becoming a habitat island for many species during decomposition, which can last for a few years to decades (Lundsten et al. 2010; Smith et al. 2015). The enormous input of organic matter from whale falls supports diverse ecosystems, rich in opportunistic species capable of tolerating the organic matter decomposition (Smith and Baco 2003; Gaudron et al. 2010). Still, as with vents and seeps, these ecosystems can have unique and specialized species and communities (Smith and Baco 2003; Braby et al. 2007; Lundsten et al. 2010; Smith et al. 2015).

The study of these communities revealed some remarkable new species, including the annelid genus *Osedax* Rouse, Goffredi & Vrijenhoek, 2004 (Siboglinidae), a group of organisms specialized to exploit bones and teeth of dead marine vertebrates (Rouse and Goffredi 2023). *Osedax* taxa lack a digestive system and have a particular endosymbiotic relationship with heterotrophic bacteria hosted in the branching root system that excavates the bone. The symbiotic bacteria are capable of metabolizing complex carbon compounds within the bones yielding nutrition to both symbionts and the *Osedax* hosts (Goffredi et al. 2005, 2007; Katz et al. 2010; Tresguerres et al. 2013). *Osedax* specimens also usually exhibit a marked sexual dimorphism, in which the females can reach 1–10 cm and the males are microscopic dwarfs (paedomorphic) and live in harems attached to the female trunk (Rouse et al. 2004, 2008; Vrijenhoek et al. 2008, 2009). The genus currently has 33 described species in all the ocean basins, with a wide bathymetric range, varying from 21 to 4204 meters (Rouse et al. 2018; Fujiwara et al. 2019; Eilertsen et al. 2020; Georgieva et al. 2023; Berman et al. 2024). More than half of the named species were first collected from the California margin, where whale-fall studies are most numerous and long-standing (Smith et al. 2015).

As in most deep-sea exploration areas of knowledge, the data on whale-fall associated species in the Global South is still limited, and it is predicted that many hundreds of whale-fall species remain to be discovered in these ocean regions (Smith et al. 2015). This prediction relies on the high abundance of large marine mammals in this region (Laws 1977) and their seasonal migratory routes between high-latitude feeding grounds and low-latitude breeding grounds (Dawbin 1966). This knowledge gap is particularly pronounced for *Osedax* species, as *Osedaxbraziliensis* Fujiwara, Jimi, Sumida & Kitazato, 2019 is the only described species for the South Atlantic Ocean (excluding Antarctic and subantarctic species) and it was found inhabiting the first reported natural whale carcass in the deep Southwest Atlantic Ocean at 4204 m depth during an expedition with the human-occupied submersible Shinkai 6500 in 2018 (Fujiwara et al. 2019).

Aiming to reduce the knowledge gap regarding the organic falls community diversity in the South Atlantic Ocean and its global connectivity, the project BioSuOr (“Biodiversity and connectivity of benthic communities in organic substrates in the deep southwest Atlantic”) was conducted between 2016 and 2017, implanting mammalian bones and wood samples in Brazilian deep waters. These samples were implanted at multiple sites across three different depths: 550, 1500 and 3300 m. The deployment of the organic falls was achieved using free-fall landers equipped with acoustic releases for recovery and were subsequently colonized by a variety of polychaetes, crustaceans and mollusks. Some of the results from this project in the Southwest Atlantic Ocean have been documented (Shimabukuro and Sumida 2019; Shimabukuro et al. 2019, 2022; Souza et al. 2021; Avila et al. 2023; Bergamo et al. 2024), reporting the occurrence of 24 animal species (Shimabukuro et al. 2022), including potential new species.

**Figure 1.**
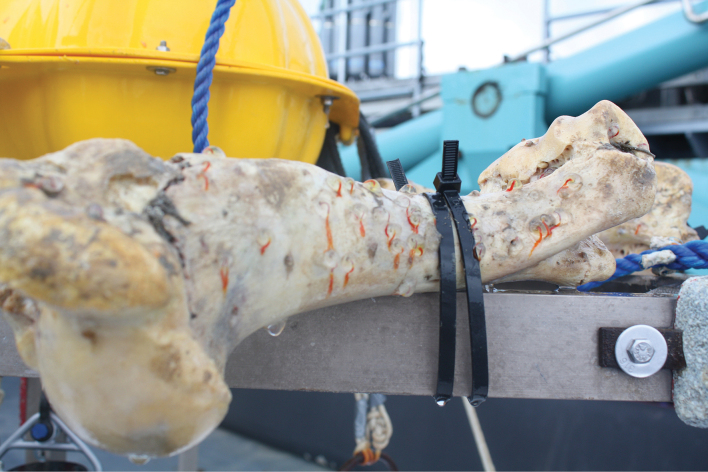
Cow femur colonized by *Osedax* individuals in an implanted free-fall lander after recovery.

During the sampling of BioSuOr project material, numerous *Osedax* specimens were observed colonizing the implanted cow bones (Fig. 1). Four *Osedax* species were recovered from this project and their mitochondrial cytochrome *c* oxidase subunit I (COI) sequences were reported and lodged on GenBank as BioSuOr-1, 2, 3, and 4 (Shimabukuro and Sumida 2019). Here, we use molecular phylogenetic and morphological data to formally describe *Osedax* ‘BioSuOr-4’, which was recovered on bones at a depth of 550 m, along with an assessment of its intraspecific diversity. Additionally, we documented the occurrence of dwarf males in this species.

## ﻿Materials and methods

### ﻿Sample collection and morphological analysis

**Figure 2.**
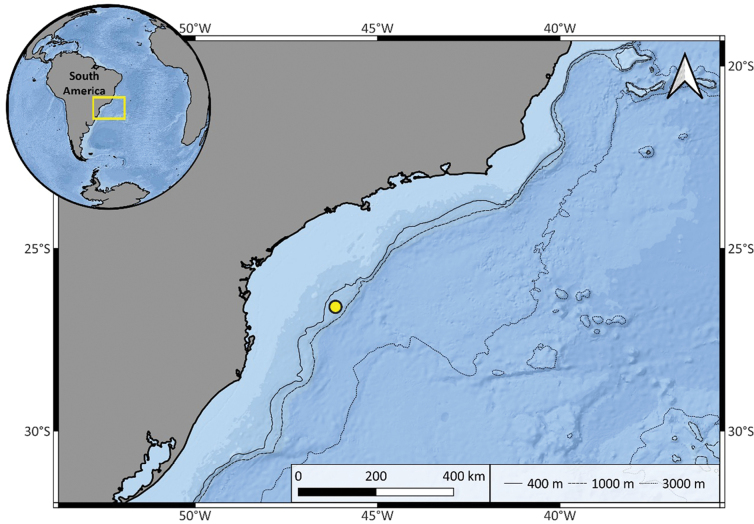
The yellow circle indicates exact position of the lander at 550 m depth off the Brazilian continental margin recovered in May 2017 with the R/V Alucia.

*Osedax* specimens investigated in this study were obtained through an in-situ experiment that involved implanting bovine bones using experimental autonomous structures (landers) equipped with acoustic releases (see Saeedi et al. 2019) in the Southwest Atlantic Ocean, off the Brazilian continental margin (26°36'13.44"S, 46°09'9.29"W) at 550 m depth (Fig. 2). Landers were deployed in July 2016 using the R/V Alpha Crucis and recovered in May 2017 with the R/V Alucia. Once onboard bones were placed in sea water at 4 °C to photograph living *Osedax*. Bones with worms were then fixed in 96% ethanol. *Osedax* specimens were later extracted at Laboratório de Mar Profundo (**LAMP**), Instituto Oceanográfico, Universidade de São Paulo, with 115 female specimens and tubes extracted using a stereomicroscope. The holotype and attached dwarf males were imaged from fixed material with Leica MZ12.5 (+ Canon Rebel T6i camera), or Leica M205C (+ Leica MC170HD camera) stereomicroscopes. A dwarf male was imaged with a Leica DMR compound microscope and Canon Rebel T6i camera.

For scanning electron microscopy (SEM) analysis, female specimens were dehydrated in absolute ethanol. They were then rinsed in two 10-min baths in a solution consisting of 50% ethanol and 50% Hexamethyldisilazane (HMDS), followed by an additional two 10-min baths in HMDS alone. Specimens were left to dry overnight, then mounted on stubs using carbon adhesive tape, sputter-coated with gold, and examined and photographed under a Zeiss Sigma VP SEM at Laboratório de Microscopia Eletrônica, Instituto de Biociências, Universidade de São Paulo.

### ﻿DNA preparation, amplification, and sequencing

DNA of 37 female specimens was extracted from their root regions using Zymo Research DNA-tissue miniprep kits, following the manufacturer’s provided protocol. Extracted DNA was utilized as template for the polymerase chain reaction (PCR) amplification of fragments of mitochondrial cytochrome *c* oxidase subunit I (COI) and 16S rRNA (16S) genes, and the nuclear 18S rRNA (18S), 28S rRNA (28S) and Histone H3 (H3) genes, using primers shown in Table 1. COI was sequenced for all specimens initially for species delimitation. Subsequently, other markers were sequenced from a single representative specimen.

**Table 1.** List of genes, primers and PCR temperature profiles used in the present study.

Gene	Primer set	Reference	Cycle
Cytochrome *c* oxidase subunit I (COI)	OsCO1r/OsCO1f	Glover et al. 2005	120s at 95 °C, 35 cycles of 60s at 94 °C, 60s at 50 °C and 60s at 72 °C, 420s at 72 °C
16S rRNA (*16S*)	16SarL/16SbrH	Palumbi 1996	180s at 95 °C, 35 cycles of 40s at 95 °C, 40s at 50 °C and 50s at 72 °C, 300s at 72 °C
18S rRNA (*18S*)	18S-1F/18S-5R	Giribet et al. 1996	180s at 95 °C, 40 cycles of 30s at 95 °C, 30s at 50 °C and 90s at 72 °C, 480s at 72 °C
	18S-a2.0/18S-9R	Giribet et al. 1996; Whiting et al. 1997	180s at 95 °C, 40 cycles of 30s at 95 °C, 30s at 50 °C and 90s at 72 °C, 480s at 72 °C
	18S-3F/18S-bi	Giribet et al. 1996; Whiting et al. 1997	180s at 95 °C, 40 cycles of 30s at 95 °C, 30s at 52 °C and 90s at 72 °C, 480s at 72 °C
28S rRNA (*28S*)	D1F/D3R	Brown et al. 1999	180s at 94 °C, 35 cycles of 60s at 94 °C, 30s at 55 °C and 110s at 72 °C, 240s at 72 °C
Histone H3 (*H3*)	H3F/H3R	Colgan et al. 1998	180s at 95 °C, 40 cycles of 30s at 95 °C, 45s at 53 °C and 45s at 72 °C, 300s at 72 °C

PCR amplification was conducted using a mixture consisting of 12.5 µl Apex^TM^ 2.0× Taq Red DNA polymerase Master Mix (Genesee Scientific), 1 µl of each appropriate forward and reverse primers (10 µM), 8.5 µl of ddH2O, and 2 µl of eluted DNA. PCR cycling was conducted in a thermal cycler following specific profiles and temperatures for each primer, as indicated in Table 1. Following confirmation of appropriate bands via gel electrophoresis, PCR products were purified using ExoSAP-IT following the manufacturer’s protocol, and sent to Eurofins Genomics Company (Louisville, Kentucky, USA) for sequencing. Resulting sequences were assembled and edited in Geneious Prime R11.5.1 (Kearse et al. 2012) before deposition in GenBank under accession numbers shown in Table 2.

**Table 2.** List of species and GenBank accession numbers for sequences in this study. New sequences in bold.

Taxa	Source/Authority	COI	16S	18S	28S	H3
**Outgroup**						
*Lamellibrachiacolumna*	Webb 1969	DQ996645	FJ347646	FJ347679	MG264417	FJ347696
*Riftiapachyptila*	Jones 1981	KP119562	KP119573	KP119591	KP119582	KP119555
*Sclerolinumbrattstromi*	Webb 1964	FJ347644	FJ347644	FJ347680	FJ347677	FJ347697
***Osedax***						
*O.antarcticus*	Glover et al. 2013	KF444422	KF444418	KF444420	–	–
*O.* ‘BioSuOr-1’	Shimabukuro and Sumida 2019	MH616036	–	–	–	–
*O.* ‘BioSuOr-2’	Shimabukuro and Sumida 2019	MH616081	–	–	–	–
*O.* ‘BioSuOr-3’	Shimabukuro and Sumida 2019	MH616075	–	–	–	–
*O.nataliae* sp. nov.	This study; Shimabukuro and Sumida 2019	MH616012–MH616016, **PP765811**–**PP765827, PP982821**–**PP982840**	**PP669598**	**PP669599**	**PP669600**	**PP766874**
*O.bozoi*	Berman et al. 2024	ON357627	ON261606	ON261611	ON261610	ON254806
*O.braziliensis*	Fujiwara et al. 2019	LC381421	–	LC381424	–	–
*O.bryani*	Rouse et al. 2018	KP119563	KP119574	KP119597	KP119584	KP119561
*O.byronbayensis*	Georgieva et al. 2023	OQ801427	OQ820973	OQ803227	–	–
*O.craigmcclaini*	Berman et al. 2024; McClain et al. 2019	MN258704	ON217799	ON220153	ON226742	ON254807
*O.crouchi*	Amon et al. 2014	KJ598038	KJ598032	KJ598035	–	–
*O.deceptionensis*	Taboada et al. 2015	KF444428	KF444419	KF444421	MG264418	KT860546
*O.docricketts*	Rouse et al. 2018	FJ347626	FJ347650	FJ347688	FJ347666	FJ347710
*O.estcourti*	Berman et al. 2024	ON211943	ON217536	ON220129	ON220739	ON254809
*O.fenrisi*	Eilertsen et al. 2020	MT556178	–	MT556473	–	–
*O.frankpressi*	Rouse et al. 2004	FJ347607	FJ347658	FJ347682	FJ347674	FJ347705
*O.jabba*	Rouse et al. 2018	FJ347638	FJ347647	FJ347693	FJ347676	FJ347703
*O.japonicus*	Fujikura et al. 2006	FM998111	–	FM995535	–	–
*O.knutei*	Rouse et al. 2018	FJ347635	FJ347648	FJ347692	FJ347664	FJ347700
*O.lehmani*	Rouse et al. 2018	DQ996634	FJ347660	FJ347689	FJ347672	FJ347706
*O.lonnyi*	Rouse et al. 2018	FJ347643	FJ347651	FJ347695	FJ347663	FJ347699
*O.* ‘MB16’	Salathé and Vrijenhoek 2012	JX280613	KP119581	KP119592	KP119588	KP119560
*O.* ‘mediterranea’	Taboada et al. 2015	KT860548	KT860551	KT860550	KT860549	KT860547
*O.mucofloris*	Glover et al. 2005	AY827562	–	–	AY941263	–
*O.nordenskjoeldi*	Amon et al. 2014	KJ598039	KJ598033	KJ598036	–	–
*O.packardorum*	Rouse et al. 2018	FJ347629	FJ347661	FJ347690	FJ347673	FJ347707
*O.priapus*	Rouse et al. 2015	KP119564	KP119575	KP119594	KP119585	KP119556
*O.randyi*	Rouse et al. 2018	FJ347615	FJ347659	FJ347684	FJ347675	FJ347712
*O.rogersi*	Amon et al. 2014	KJ598034	KJ598037	KJ598040	–	–
*O.roseus*	Rouse et al. 2008	FJ347609	FJ347657	FJ347683	FJ347670	FJ347709
*O.rubiplumus*	Rouse et al. 2004	EU852488	FJ347656	FJ347681	FJ347671	FJ347704
*O.ryderi*	Rouse et al. 2018	KP119563	KP119574	KP119597	KP119584	KP119561
*O.* ‘sagami-3’	Pradillon et al. unpublished	FM998081	–	FM995537	–	–
*O.* ‘sagami-4’	Pradillon et al. unpublished	FM998082	–	FM995541	–	–
*O.* ‘sagami-5’	Pradillon et al. unpublished	FM998083	–	FM995539	–	–
*O.sigridae*	Rouse et al. 2018	FJ347642	FJ347655	FJ347694	FJ347669	FJ347711
*O.talkovici*	Rouse et al. 2018	FJ347621	FJ347654	FJ347685	FJ347668	FJ347698
*O.tiburon*	Rouse et al. 2018	FJ347624	FJ347653	FJ347687	FJ347662	FJ347702
*O.traceyae*	Berman et al. 2024	ON211990	ON212680	ON10988	ON220740	ON254808
*O.ventana*	Rouse et al. 2018	EU236218	FJ347652	FJ347686	FJ347665	FJ347701
*O.westernflyer*	Rouse et al. 2018	FJ347631	FJ347649	FJ347691	FJ347667	FJ347708
*O.waadjum*	Georgieva et al. 2023	OQ801430	OQ820974	OQ803228	–	–

### ﻿Phylogenetic analysis

Assembled sequences were align using MAFFT (Katoh and Standley 2013; Rozewicki et al. 2019) in the program Mesquite (Maddison and Maddison 2019), including sequences from 41 *Osedax* named and unnamed species and members of three other Siboglinidae genera as outgroups (Table 2).

The most appropriate evolutionary model for each marker was recovered using ModelTest-NG (Darriba et al. 2020). The best models chosen (based on AICc) were COI = GTR+I+G4, 16S = TIM2+I+G4, 18S = GTR+I+G4, 28S = TIM3+I+G4, and H3 = TVMef+I+G4. A maximum likelihood (ML) phylogenetic tree using the concatenated sequences of all five markers was generated using the RAxML GUI program (Edler et al. 2021). Node support was assessed through bootstrapping with 1000 replicates. We chose not to conduct a Bayesian phylogenetic analysis of the data as it would yield very similar estimate of the molecular phylogeny as the maximum likelihood results. Also, as pointed out in Berman et al. (2024), missing data for many *Osedax* terminals is likely responsible for the lack of well-supported relationships rather than any particular analytical method.

Minimum genetic distance based on uncorrected *p*–distance of COI was calculated using PAUP* (Swofford 2002) between the sampled specimens and the other 44 sequences from reported *Osedax* and non-*Osedax* species. These distances were calculated using the COI alignment employed in the phylogenetic analyses.

To provide insights into the genetic relationships at the population level of the new *Osedax* species described here, a TCS haplotype network (Clement et al. 2000) using a 426 bp alignment of the COI marker was recovered for the 37 specimens sequenced using the program PopART (Leigh and Bryant 2015).

## ﻿Results

### ﻿Taxonomy

**Siboglinidae Caullery, 1914**

***Osedax* Rouse, Goffredi & Vrijenhoek, 2004**

#### ﻿ Osedax nataliae

Taxon classificationAnimaliaSabellidaSiboglinidae

Gularte, Sumida, Bergamo & Rouse sp. nov

D7882582-9664-513B-969E-710A5A93D409

https://zoobank.org/ED3DE09A-776C-46FF-8418-59881D751365

Figs 3 , 4 , 5

Osedax ‘BioSuOr-4’ sec. Shimabukuro and Sumida 2019.

##### Type material.

***Holotype***: MZUSP 6201, Female, preserved in ethanol, derived from an experimentally deployed cow bones (*Bostaurus*) at a depth of 550 m, collected with *R/V Alucia* on the continental margin off São Paulo state, Brazil (26°36'13.44"S, 46°09'9.29"W) on 18 May 2017. ***Paratypes***: MZUSP 6203–6204, all females (30), preserved in ethanol, collected on cow bones deployed at the same locality and date as the holotype. Two dwarf male (allotypes), fixed in ethanol from tube of holotype: MZUSP 6202, same date and locality as holotype.

##### Diagnosis and description.

**Figure 3.**
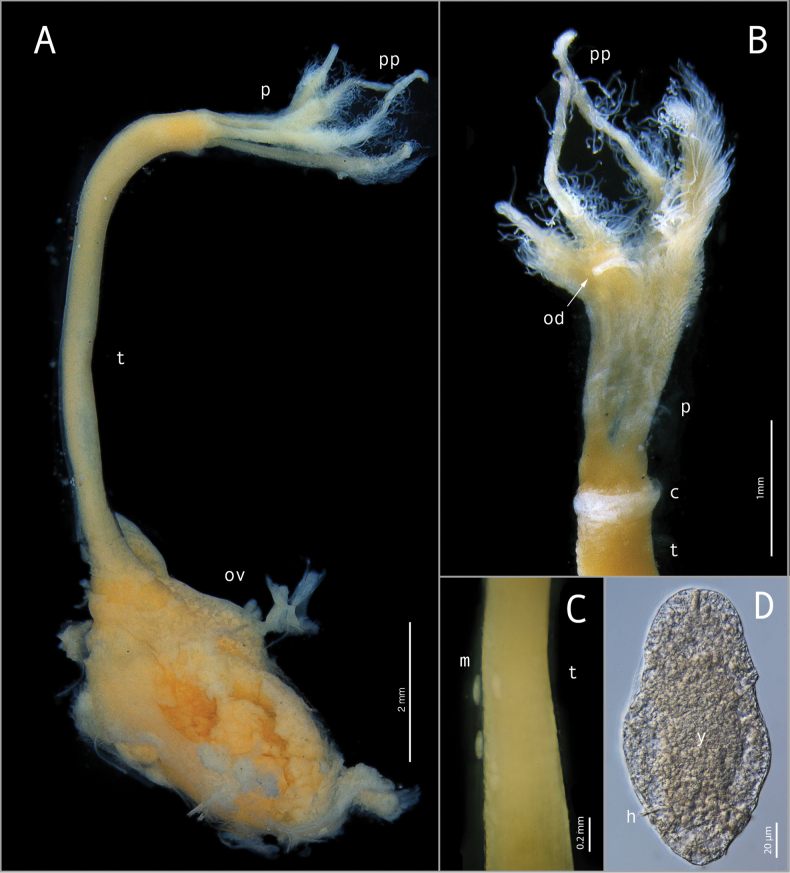
*Osedaxnataliae* sp. nov. Preserved female holotype (MZUSP 6201) **A–C** and male specimens (MZUSP 6202) **D**: **A** lateral view of the entire specimen **B** detail of palps and trunk **C** detail of trunk with male attached to the surface **D** light microscope of individual male (preserved). Abbreviations: c, collar; m, male; od, oviduct; ov, ovisac; p, palps; pp, pinnules; t, trunk; h, hooks; y, yolk.

**Figure 4.**
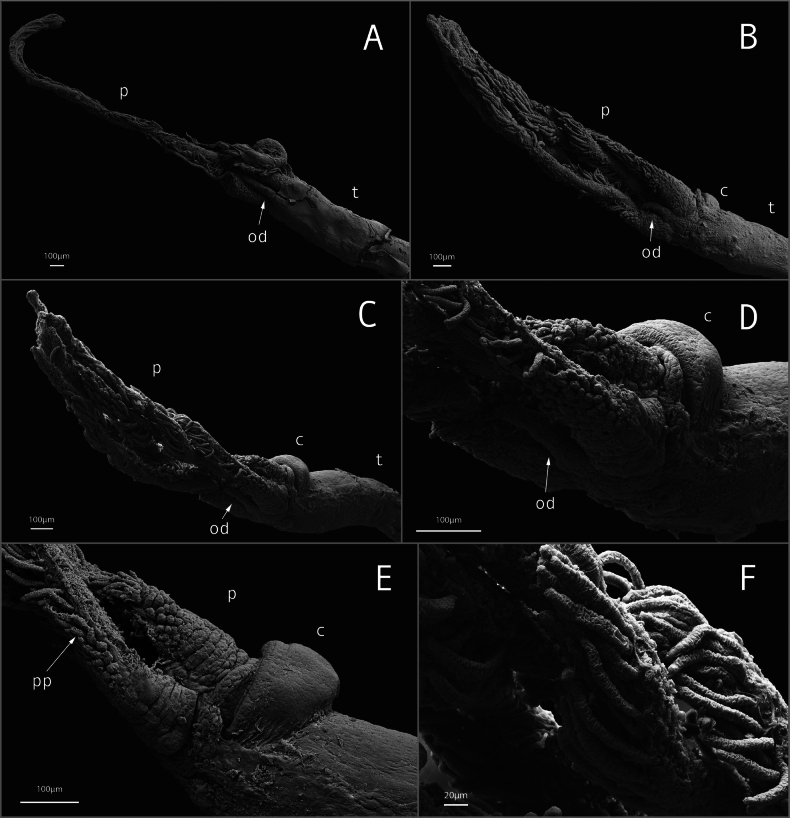
*Osedaxnataliae* sp. nov. Scanning Electron Microscopy (SEM) of two paratypes. Paratype (MZUSP 6204) **A** dorsal view of palps and trunk end of paratype 1. Paratype (MZUSP 6205) **B** dorsal view of palps and trunk end **C** lateral view highlighting oviduct and collar position **D** detail of the base of the palps **E** lateral view of the collar **F** detail of the pinnules in the palps. Abbreviations: c, collar; od, oviduct; p, palps; pp, pinnules; t, trunk.

**Figure 5.**
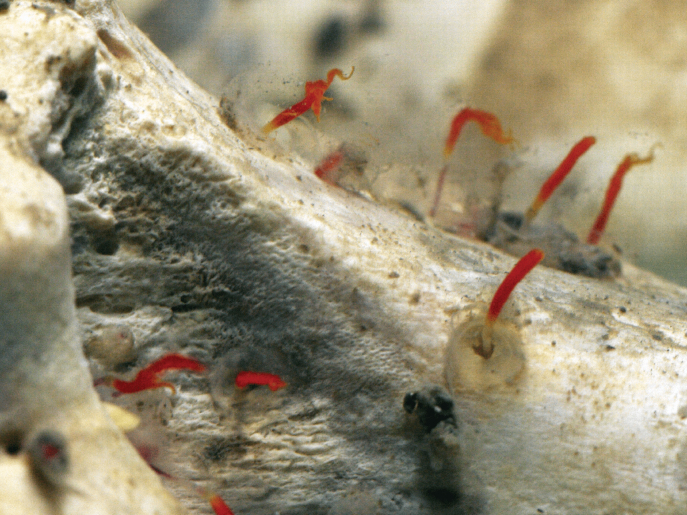
*Osedaxnataliae* sp. nov. Live specimens photographed alive onboard R/V Alucia after recovery from the lander. Red palps and trunk are partially extended in gelatinous tubes.

Holotype female (Fig. 3A–C); body length ~ 14.7 mm; gelatinous tube (removed) 0.2 wide, longer than trunk and crown; crown of palps, ~ 2.8 mm long; trunk length ~ 7.4 mm, width ~ 0.5 mm; root structure ~ 4.5 mm long; width ~ 2.4 mm; Crown of four pinnulated palps, with the pinnules arranged along the outer margin of the palps (Figs 3B, 4). Conspicuous oviduct shorter than palps (Figs 3B, 4A–D). Collar ventrally along the margin of anterior trunk, except for the dorsal portion (Figs 3B, 4B–E). Live specimens with palps bright red-orange distally, becoming yellow and then white proximally to the boundary with the trunk (Fig. 5). No obvious pigmentation on trunk or demarcation into upper and lower trunk. Root structure missing in holotype, bulbous or lobulate in paratypes. Ovisac an ellipsoidal mass contain oocytes at various stages of development (Fig. 3A). Dwarf male ~ 170 μm in length, fusiform, no appendage organs (Fig. 3C); posterior hooks present (Fig. 3D).

##### Distribution.

Known from the continental margin off São Paulo state, Santos basin, Brazil, at a depth of 550 m; on experimentally deployed cow bones.

##### Molecular results.

**Figure 6.**
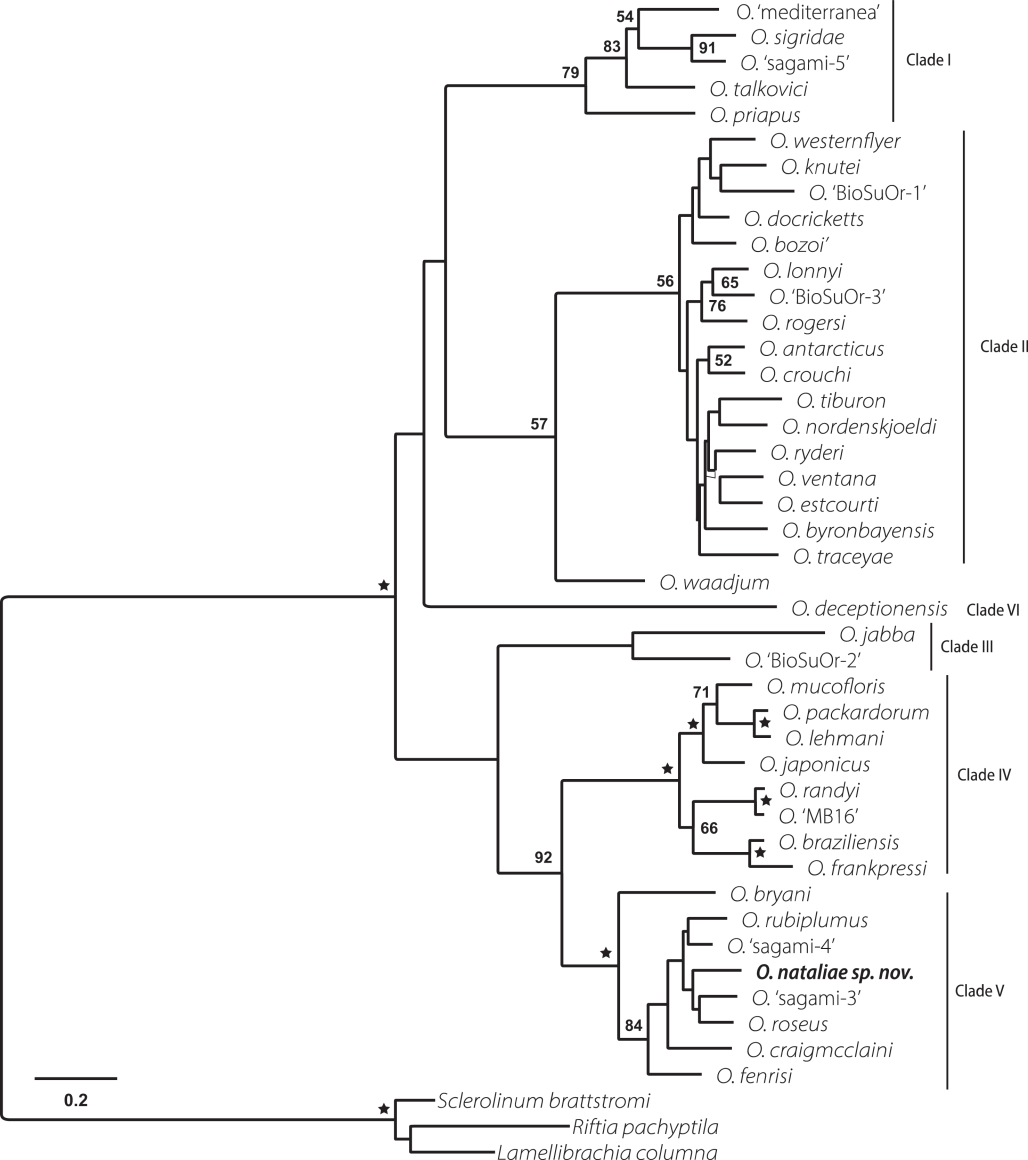
*Osedax* phylogenetic analysis. Maximum likelihood phylogenetic tree based on a partitioned concatenated dataset of COI, 16S, 18S, 28S, and H3 markers (MAFFT-aligned) for the data shown in Table 2. Bootstrap support values are indicated. Black star values were ≥ 95% (BS). Missing values indicate BS < 50%.

**Figure 7.**
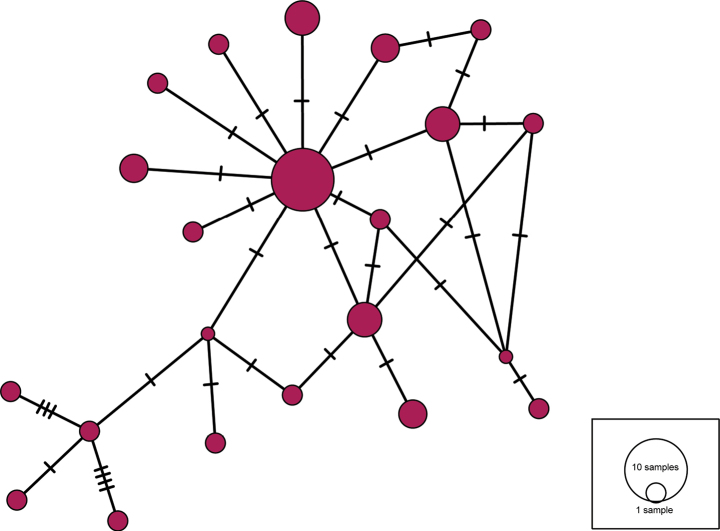
Haplotype network using COI for 37 *Osedaxnataliae* sp. nov. Circles are haplotypes and crosshatches are single nucleotide substitutions.

The final lengths of sequences for the different genetic markers were 482–600 bp (COI), 454 bp (16S), 1769 bp (18S), 997 bp (28S) and 309 bp (H3). Uncorrected intraspecific divergence of *O.nataliae* sp. nov. for COI was up to 2.44%. In terms of distance, the most closely related species to *O.nataliae* sp. nov. was *O.* ‘sagami-3’, with a minimum interspecific distance for COI of 13.92% (Suppl. material 1). The phylogenetic analysis of the concatenated dataset of the five markers placed *Osedaxnataliae* sp. nov. in the well-supported Clade V (see Rouse et al. 2018) although relationships within the clade were poorly supported. The new species was recovered as sister species to the clade formed by *O.* ‘sagami-3’, known from NW Pacific at unknown depth, and *Osedaxroseus*, from NW and NE Pacific at depths of 633 to 1820 m (Fig. 6). A total of 22 distinct haplotypes were recovered for the COI dataset (*n* = 38), with the most common one being shared by ten individuals (Fig. 7). Despite originating from a single experimental lander, the network reveals a central and more common haplotype surrounded by several closely related and some more distant haplotypes with numerous nucleotide substitutions.

##### Remarks.

*Osedaxnataliae* sp. nov. is part of the Clade V according to the phylogenetic analysis (Fig. 6) and shares some important morphological features with the other taxa within this clade, such as pinnules inserted on the outer margin of palps (Fig. 4D, E) and a collar at the base of the crown (Figs 4B, 5B–E). The collar of *Osedaxnataliae* sp. nov. and *Osedaxroseus* (the closest species in molecular phylogeny that has a morphological description) are similar in shape and position, though more inflated in *O.nataliae* sp. nov. Some specimens of *Osedaxnataliae* sp. nov. appear to lack a collar, which could be an artifact of fixation. The dwarf males of *O.nataliae* sp. nov., with a length of 170 μm, are notably smaller than the males of *O.rubiplumus* (400 μm–1.1 mm long) but similar in size to those of *O.roseus* (130–210 μm) and *O.frankpressi* (150–250 μm). The body size (length of crown + trunk) of *Osedaxnataliae* sp. nov. females varied markedly among the individuals examined, ranging from 4 mm to 15 mm, with a mean value of 6.76 mm. When compared with groups from the same clade, the body size is like *O.roseus*, *O.bryani*, and *O.fenrisi* females but much smaller than *O.rubiplumus*. *Osedaxnataliae* sp. nov. is not obviously distinguishable from its relatives on morphology. Its notable features such as the red-orange distal crown of pinnulate palps, yellowing towards the base, collar, and long trunk (Fig. 3) may occur in other species of Clade V, such as *O.roseus*. However, molecular data from both the phylogenetic analysis (Fig. 6) and COI distance (Suppl. material 1) confirm *Osedaxnataliae* sp. nov. as a new species.

##### Etymology.

This species is named after Natalia Gularte, mother of the first author, in recognition of her long and continued support in this research effort.

## ﻿Discussion

This study formally describes a second species of *Osedax* from the South Atlantic Ocean, combining both morphological and molecular approaches. *Osedaxnataliae* sp. nov. was previously reported under the informal epithet of ‘BioSuOr-4’ (see Shimabukuro and Sumida 2019) based on COI alone though its phylogenetic placement was not clear based on such limited data. With the additional data of five molecular markers, *O.nataliae* sp. nov. joins *O.bryani*, *O.fenrisi*, *O.craigmcclaini*, *O.rubiplumus*, *O.* ‘sagami-4’, *O.roseus*, and *O.* ‘sagami-3’, to supplement the membership of the well-supported Clade V (Vrijenhoek et al. 2009; Rouse et al. 2015, 2018; Eilertsen et al. 2020; Berman et al. 2024). However, relationships within clade V are poorly supported likely owing to the lack of DNA data for most of the molecular markers used here for terminals such as *O.* ‘sagami-3’, *O.* ‘sagami-4’, and *O.fenrisi*. The present results suggest *O.nataliae* sp. nov. is nested among a Pacific clade of *Osedax* (Fig. 5), but sampling of *Osedax* diversity is presently biased towards that ocean basin.

The migratory routes of many species of whales through the Atlantic Ocean, including the sub-Atlantic populations of humpback whale (*Megapteranovaeangliae*) which migrate from South Georgia Islands through Rio Grande Rise and northwards to Abrolhos Bank (Best et al. 1993; Zerbini et. al 2006; Santos et al. 2010; Wedekin et al. 2014) suggest that the deep-sea oceans in the Southern Hemisphere may provide a rich supply of whale carcasses to support *Osedax* species. The report of 16 whale-fall-associated new species, including four *Osedax* species from the BioSuOr project conducted in the Southwest Atlantic Ocean (Shimabukuro et al. 2022) supports this hypothesis.

The haplotype network for *O.nataliae* sp. nov. (Fig. 7) reveals high genetic diversity within the study area, with 22 haplotypes recovered from the 37 specimens sequenced. This network also shows no evidence of any population ‘bottleneck’, which would have been indicated by a much lower diversity of haplotypes for the number of specimens sequenced (Avise 2000). This diverse haplotype network aligns with observations seen previously in other *Osedax* species (Glover et al. 2005; Rouse et al. 2008; Amon et al. 2014).

As part of the BioSuOr project, *O.nataliae* sp. nov. was exclusively reported from cow bones deployed at a depth of 550 m. No information is available regarding the possibility of this species colonizing only small bones nor is there data on its depth range capability. Further research is needed to investigate the potential substrate preferences and depth range of *O.nataliae* sp. nov. to better understand its ecological niche and distribution in deep-sea ecosystems.

## Supplementary Material

XML Treatment for Osedax nataliae

## ﻿Additional information

### Author ORCIDs

Thammy Gularte https://orcid.org/0000-0002-3121-1973

Paulo Y. G. Sumida https://orcid.org/0000-0001-7549-4541

Gilberto Bergamo https://orcid.org/0000-0001-5464-909X

Greg W. Rouse https://orcid.org/0000-0001-9036-9263

### Data availability

All data generated during this study are included in this article. Sequences are published in GenBank and BOLD.

## Supplementary materials

Supplementary material 1 Minimum genetic distance based on uncorrected p–distance between species within Clade V Data type xlsx Explanation note The maximum intraspecific divergences among *Osedaxnataliae* sp. nov. specimens are in bold. https://binary.pensoft.net/file/1189243This dataset is made available under the Open Database License (http://opendatacommons.org/licenses/odbl/1.0/). The Open Database License (ODbL) is a license agreement intended to allow users to freely share, modify, and use this Dataset while maintaining this same freedom for others, provided that the original source and author(s) are credited.Thammy Gularte, Paulo Y. G. Sumida, Gilberto Bergamo, Greg W. Rouse
